# A Systematic Review of Vascular Injuries: A Review of Petechiae, Purpura, and Ecchymosis in Critical Situations Following COVID‐19 Vaccination

**DOI:** 10.1002/hsr2.70503

**Published:** 2025-03-12

**Authors:** Yasamin Kalantari, Seyed Mohamad Sadegh Mirahmadi, Sanam Alilou, Sara Sadeghi, Zeinab Aryanian, Alireza Jafarzadeh, Azadeh Goodarzi

**Affiliations:** ^1^ Department of Dermatology, Razi Hospital Tehran University of Medical Sciences, Razi Hospital, Vahdat‐e‐Eslami Square Tehran Iran; ^2^ School of Medicine Iran University of Medical Sciences Tehran Iran; ^3^ Student Research Committee, Faculty of Medicine Iran University of Medical Sciences Tehran Iran; ^4^ Division of Dermatology, Department of Pediatrics, Alberta Children's Hospital University of Calgary Calgary AB Canada; ^5^ Rasool Akram Medical Complex Clinical Research Development Center (RCRDC) Iran University of Medical Sciences Tehran Iran; ^6^ Autoimmune Bullous Diseases Research Center Tehran University of Medical Sciences Tehran Iran; ^7^ Department of Dermatology Babol University of Medical Sciences Babol Iran; ^8^ Department of Dermatology, Rasool Akram Medical Complex Clinical Research Development Center (RCRDC), School of Medicine Iran University of Medical Sciences (IUMS) Tehran Iran; ^9^ Department of Dermatology Rasool Akram Medical Complex Tehran Iran

**Keywords:** COVID‐19, Moderna, mRNA‐1273, Oxford‐AstraZeneca, Pfizer‐BioNTech, SARS‐CoV‐2

## Abstract

**Background and Aims:**

Vascular injuries characterized by petechiae, purpura, and ecchymosis have been reported as potential adverse effects following COVID‐19 vaccination. This study aims to identify the characteristics of patients experiencing vascular injuries postvaccination and to outline key clinical considerations.

**Methods:**

A systematic review was conducted in accordance with PRISMA guidelines. A comprehensive search of Scopus, Web of Science, and PubMed/MEDLINE databases was performed for English‐language publications up to July 2024. Eligible studies included reports of vascular injuries following COVID‐19 vaccination.

**Results:**

Of the 1064 articles retrieved, 35 studies met the inclusion criteria. The majority of cases presented symptoms after receiving the first doses of Pfizer‐BioNTech, Moderna, AstraZeneca, and Janssen vaccines. Diagnosed conditions included thrombotic thrombocytopenic purpura (TTP), immune thrombocytopenic purpura (ITP), vasculitis, and acquired hemophilia A. None of the patients tested positive for SARS‐CoV‐2 at the time of diagnosis. The most commonly affected sites were the lower extremities, with petechiae, purpura, and ecchymosis being the predominant manifestations.

**Conclusion:**

Our findings suggest a possible but unconfirmed association between COVID‐19 vaccination and the development of vascular injuries, including petechiae, purpura, and ecchymosis. These symptoms may serve as early indicators of critical conditions requiring urgent medical intervention. Further research and postvaccination monitoring are necessary to establish causality and assess potential risk factors.

## Summary

**What Is Already Known About This Topic?**

- COVID‐19 vaccines have been associated with a range of cutaneous adverse effects, including maculopapular, morbilliform, and urticarial rashes, as well as more severe manifestations like leukocytoclastic vasculitis, pityriasis rosea‐like lesions, and specific cases of petechiae, purpura, and ecchymosis.
- The development of petechiae, purpura, and ecchymosis following vaccination could be attributed to multiple factors, such as trauma, infection, or hematological conditions like ITP, TTP, and DIC. These conditions have been documented after vaccinations for other viral diseases, indicating a possible pattern.

**What Does This Study Add?**

- The systematic review initially identified 1064 articles, ultimately selecting 35 relevant studies on vascular injuries. These studies included a nearly equal number of male (48.57%) and female (51.42%) patients, with a mean age of 55.4 years.
- Most cases of vascular injuries were associated with the first doses of Pfizer‐BioNTech (52.77%), Moderna (22.22%), AstraZeneca (22.22%), and Janssen (2.77%). Symptoms varied in onset from less than 1 day to 3 weeks postvaccination, and patients were diagnosed with conditions such as TTP, ITP, vasculitis, and acquired hemophilia A.
- Cutaneous symptoms, including petechiae (48.57%), purpura (31.42%), and ecchymosis (17.14%), were notably prevalent, especially on the lower extremities.

## 1 Introduction

After the emergence of the COVID‐19 pandemic in 2019, significant efforts were made to develop vaccines against the virus. With the introduction of COVID‐19 vaccines, various cutaneous adverse effects have been reported. These cutaneous manifestations range from maculopapular, morbilliform, and urticarial rashes to leukocytoclastic vasculitis, pityriasis rosea‐like lesions, and chilblains [1, 2, 3]. Among these, petechiae, purpura, and ecchymosis are notable findings associated with COVID‐19 vaccines [4, 5, 6, 7].

These lesions may arise from several factors, including trauma, infection, vasculitis, drug reactions, or hematological conditions such as idiopathic thrombocytopenic purpura (ITP), thrombotic thrombocytopenic purpura (TTP), and disseminated intravascular coagulation (DIC). Conditions like TTP have been previously documented following vaccinations against other viral diseases, including influenza, pneumococcal infections, and rabies [2]. Notably, similar cases have also been reported in literature following COVID‐19 vaccination.

The aim of the current study is to provide a systematic review of petechiae, purpura, and ecchymosis related to COVID‐19 vaccines, particularly in critical situations. As individuals receive booster doses of COVID‐19 vaccines, it is vital for healthcare providers to be aware of potential adverse effects, especially those that may be life‐threatening.

## 2 Materials and Methods

### 2.1 Protocol and Registration

This systematic review adhered to the Preferred Reporting Items for Systematic Reviews and Meta‐Analyses (PRISMA) guidelines [8].

### 2.2 Eligibility Criteria

We included original English research articles that met the following criteria:

- (a) Reports of potentially critical vascular injuries requiring immediate medical attention, including immune thrombocytopenic purpura (ITP), thrombotic thrombocytopenic purpura (TTP), disseminated intravascular coagulation (DIC), acquired hemophilia, and vasculitis.
- (b) Hemoglobin levels below 9 g/L.
- (c) Platelet counts under 15,000/mm³.
- (d) Altered mental status.
- (e) Internal hemorrhage.
- (f) Brain hemorrhage.
- (g) Gross hematuria.
- (h) Widespread skin lesions.

Exclusion criteria:

- (a) Original articles reporting noncritical mucocutaneous bleeding manifestations, such as petechiae, purpura, or ecchymosis, without evidence of severe vascular injury.

### 2.3 Information Sources and Search Strategy

A systematic search was conducted using relevant keywords in MEDLINE (via PubMed), Web of Science, and Scopus for English publications up to July 2024. The following search terms were used:

(*post OR after OR following) AND* (*COVID‐19 OR SARS‐CoV‐2 OR coronavirus*) *AND* (*petechiae OR purpura OR purpuric OR ecchymosis OR ecchymoses OR hemorrhage OR ‘cutaneous adverse events’ OR ‘vascular injury’*) *AND* (*vaccination OR vaccine*).

### 2.4 Data Management and Selection Process

A total of 1064 articles retrieved from the database search were imported into EndNote X20 (Clarivate Analytics, Philadelphia). After removing duplicates, two authors (SA and YK) independently screened the studies based on their titles and abstracts. Eligible full‐text articles were reviewed according to the predefined inclusion and exclusion criteria. Any discrepancies in the selection or data extraction process were independently evaluated by a third researcher (AG), with disagreements resolved through consensus.

### 2.5 Data Collection Process and Data Items

The following data were extracted from each included study:

- Study characteristics: Year of publication, first author's name, type of study, and number of included participants.
- Patient demographics: Mean age, gender, past medical history, past drug history, and history of COVID‐19 infection.
- Vaccination details: Type of COVID‐19 vaccine, dose received, and interval between vaccination and symptom onset.
- Clinical findings: Cutaneous manifestations, lesion locations, associated symptoms, and non‐cutaneous findings.
- Laboratory and diagnostic data: Hematological parameters, pathology results, and final diagnoses.
- Treatment approaches and outcomes.

### 2.6 Statistical Analysis

All statistical analyses were conducted using [Specify Software, e.g., SPSS v.28 (IBM Corp., Armonk, NY) or R v.4.2.0 (R Foundation for Statistical Computing, Vienna, Austria)]. Descriptive statistics were reported as means ± standard deviations (SD), medians with interquartile ranges (IQRs), or frequencies (%), as appropriate.

- Comparative analyses between groups were performed using Student's *t*‐test or Mann–Whitney *U* test for continuous variables and *χ* ^2^ or Fisher's exact test for categorical variables, depending on data distribution.
- Pre‐specified analyses focused on the association between vaccine type and vascular injury presentation. Exploratory subgroup analyses included stratifications by age, gender, and vaccine dose.
- A priori significance levels were set at *p* < 0.05, with all tests conducted as two‐sided unless otherwise specified.
- Adherence to the SAMPL guidelines was ensured for reporting descriptive and inferential statistics.

## 3 Results

**Figure 1:**
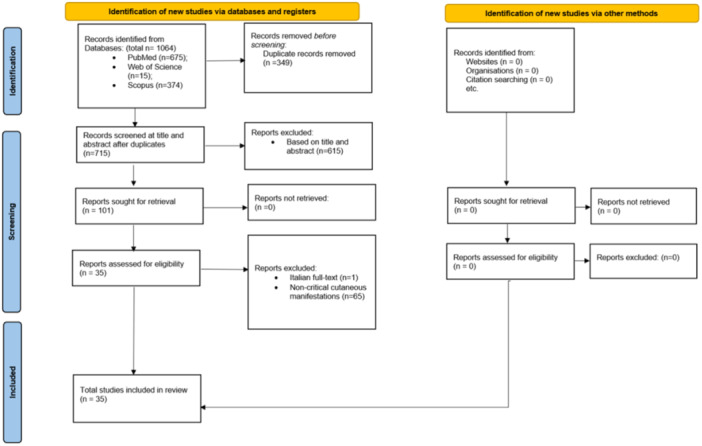
PRISMA diagram.

In total, we identified 1064 articles from three databases. After removing duplicates, 715 records remained. Our systematic review ultimately included 35 articles, as illustrated in Figure 1. This comprised 32 case reports and 3 case series, providing a comprehensive overview of vascular injuries following COVID‐19 vaccination. Table 1 summarizes the findings of these studies on vascular injuries postvaccination.

**Table 1:** Characteristics of the included studies.

Author name, year of publication	Study type, number of patients	Mean age and gender	Diagnosis	Lab data	Non‐cutaneous findings	Cutaneous findings, location of lesions, and symptoms	Type of covid‐19 vaccine, dose of covid‐19 vaccine, interval between vaccination and symptom presentation	History of COVID‐19 infection	Past medical history/past drug history	Pathologic findings	Treatment
Ai Vuen et al. [1]	Case report, 1	80, male	Acquired haemophilia A	Haemoglobin of 73 g/L (130–180), aPTT at 78.7 s (25.4–38.4), low FVIII assay of 6.7% (62.6–165.3).	Left thigh haematoma and symptomatic anaemia.	Bruising and ecchymosis on upper and lower limbs.	Pfizer‐BioNTech, first dose, 2 weeks.	No	Type 2 diabetes mellitus, hypertension, dyslipidemia, chronic kidney disease stage 3a, benign prostatic hyperplasia (BPH) and glaucoma in both eyes/calcium carbonate, alfuzosin, cardiprin, pantoprazole, atorvastatin, bisoprolol and metformin	N/A	Tranexamic acid, packed red blood cells, fresh frozen plasma. Methylprednisolone, recombinant activated FVII (rFVIIa), Azathioprine
Al Ahmad et al. [2]	Case report, 1	37, male	TTP	Hemoglobin of 83 g/L with 8% reticulocytes, a low platelet count of 14 × 10^9^/L, polychromasia, fragmented red blood cells. high lactate dehydrogenase (LDH) of 1138 IU/L, a low haptoglobin of less than 0.1 g/L, indirect hyperbilirubinemia, and negative coombs test	Dizziness, fatigue, and headache associated with exertional shortness of breath and palpitation, dark urine.	Petechia over his extremities	AstraZeneca Oxford, first dose, 10–15 days	No	N/A	N/A	Plasma exchange, corticosteroids, and rituximab.
Alislambouli et al. [3]	Case report, 1	61, male	TTP	Hemoglobin 65 g/L, platelets 6 × 10^9^/L, schistocytes, ADAMTS13 activity level of less than three	Confusion, fever, headache, emesis, dark urine	Leg ecchymosis	Pfizer, first dose, 5 days	No	None	N/A	Plasmapheresis, corticosteroids, rituximab
Bennett et al. [4]	Case report, 1	32, female	ITP	Platelet count of 1000/mL	N/A	Bruising and petechiae	Moderna, first dose, 11 days	N/A	The patient was pregnant at at 86/7 weeks of gestation	N/A	Oral prednisone
Choi et al. [6]	Case series, 3	60, male	IgA vasculitis	His urinalysis showed hematuria (RBC count 21–50/HPF) and a urine protein–creatinine ratio (UPCR) of 3902 mg/g	Abdominal pain and melena	A purpuric rash on his upper and lower extremities, abdomen, and lower back	Oxford/AstraZeneca, 10 days	N/A	None	N/A	Prednisolone
Choi et al. [6]	Case series, 3	63, male	IgA vasculitis	Gross hematuria (RBC count 11–20/HPF), proteinuria (UPCR 512 mg/g), hemoglobin level of 7.1 g/dL	Abdominal pain, arthralgia	Purpura on both lower legs	Moderna, 1 day	N/A	N/A	N/A	Prednisolone
Choi et al. [6]	Case series, 3	74, male	IgA vasculitis	Overt hematuria (RBC count over 50/HPF) and proteinuria (UPCR 4771 mg/g). hemoglobin count was 4.7 g/dL, systolic blood pressure was 70 mmHg, and pulse rate was 100 bpm.	Myalgia, arthralgia, and melena.	Purpuric rash on his extremities, abdomen	Oxford/AstraZeneca,14	N/A	Benign prostatic hyperplasia	A histopathological analysis of the ileum showed diffuse neutrophil infiltration in the submucosal layer, hemorrhaging, necrosis, and extensive fibrin deposition, which corresponded with the diagnosis of IgA vasculitis	Methylprednisolone, prednisolone after the methylprednisolone treatment ended. Azathioprine
Collins et al.[7]	Case report, 2	22, woman	ITP	Platelet count was 5000/μL	N/A	Generalized petechial rash	Pfizer BioNTech, first dose, 11 days	Negative	Attention deficit hyperactivity disorder, depression	N/A	Dexamethasone
Condorelli et al. [9]	Case report, 3	52, male	ITP	Platelet count, PLT = 1.000/mm^3^	Gum bleeding	Diffuse cutaneous purpura	AstraZeneca, first dose, 3 weeks	Negative	None	N/A	Methylprednisolone
Condorelli et al. [9]	Case report, 3	73, male	ITP	PLT = 2.000/mm^3^	N/A	Tongue and oral mucosa petechiae along with subcutaneous ecchymosis on forearms and abdomen	Pfizer‐BioNTech, second, 2 days	N/A	Hypertension, diabetes mellitus on insulin therapy, hyperlipidemia, coronary artery bypass grafting, and iron deficiency anemia	N/A	Methylprednisolone, IVIG
Cooper and Switzer [10]	Case report, 1	24, woman	ITP	Platelets of 1000	N/A	Hemorrhagic bullae of the oral mucosa and tongue and a diffuse petechial rash of the bilateral lower extremities and abdomen with scant spots on her chest and proximal upper extremities	Pfizer‐BioNTech, first, 10 days	Negative	Dysfunctional uterine bleeding secondary to her Etonogestrel implant, mild asthma controlled with as needed albuterol inhaler, and vitamin D deficiency.	N/A	Prednisone
De Bruijn et al. [11]	Case report, 1	38, female	TTP	Undetectable levels of ADAMTS13 enzyme activity	Central serous chorioretinopathy of eye	Bruising and petechiae	Pfizer BioNTech, first and second, 2 weeks	Negative	None	N/A	Methylprednisolone plasma exchange, FFP
Fu et al. [12]	Case report, 1	77, male	Acquired hemophilia A (AHA)	The activity of von Willebrand factor function was elevated (230%). low factor VIII (FVIII) activity and high titer of FVIII inhibitor. Isolated activated partial thromboplastin time (aPTT) prolongation was found.		Multiple ecchymoses at bilateral forearms and legs	Moderna, second, 3 weeks	N/A	N/A	Sub‐epidermal blistering with fibrin and abundant neutrophils. immunofluorescence study revealed linear deposition of IgG and C3 at the dermis–epidermis junction	FVIII inhibitor bypassing activity (FEIBA) of recombinant factor VII activated (rFVIIa) prednisolone Oral cyclophosphamide
Ghosh et al. [13]	Case report, 1	63, female	ITP	Platelet count was 0/μL. the patient tested positive for Sjogren's Ab (SS‐A) and scleroderma antibodies	Dyspnea	Generalized petechiae and subcutaneous bruises on the lower back	Pfizer BioNTech, second, 1	N/A	COPD, type 2 diabetes mellitus, and hypertension, hypoglycemic agents, antihypertensives, and citalopram	N/A	Dexamethasone, IVIG
Giuffrida et al. [14]	Case report, 2	83, female	TTP	Hemoglobin 5.6 g/dL, platelet count 23 × 10^9^/L, increased reticulocytes, increased lactate dehydrogenase (1905 U/L, normal values [n.v:] 0–248), increased unconjugated bilirubin (5.5 mg/dL, n.v: 0.30–1.20) and reduced haptoglobin ( < 7 mg/dL), increased number of schistocytes (10% per field). Markedly reduced activity (below 10%) with a high titer of anti‐ ADAMTS13 antibodies	N/A	Diffuse petechiae and venipuncture hematomas	Pfizer‐BioNTech, first, first, 1 week	N/A	Undifferentiated connective tissue disease treated with low‐dose steroids and steroid‐induced diabetes mellitus	N/A	Methylprednisolone, plasma exchange in humanized anti von Willebrand factor nanobody, aplacizumab.
Giuffrida et al. [14]	Case report, 2	30, female	TTP	Hemoglobin 8.9 g/dL, platelet count 11 × 10^9^/L, presence of schistocytes (5%–10% per field), reduced activity (below 10%), while a high titer of anti‐ADAMTS 13 antibodies	Headache and fatigue	Diffuse petechiae	Pfizer‐BioNTech, first, 18 days	N/A	b‐Thalassemia carrier	N/A	Plasma exchange in caplacizumab and intravenous methylprednisolone
Helms et al. [15]	Case report, 1	74, male	ITP	Platelet count of 10 × 10^9^/L	Acute epistaxis, progressive, generalized weakness, back pain causing inability to ambulate, urinary retention, constipation and encephalopathy with dysarthria.	Diffuse cutaneous purpura	Moderna, first, a few hours	Negative	Hypertension, gout, hyperlipidemia and nonischemic cardiomyopathy	N/A	Dexamethasone, platelet transfusions, rituximab eltrombopag, IVIg
Hung et al. [16]	Case report, 1	29, male	TTP	Thrombocytopenia (platelet count 42 × 10^9^/L), elevated d‐dimer level ( > 10,000 FEU ng/mL), and positive anti‐PF4 IgG antibody ELISA tests [658.3 ng/mL (normal, 42.1–313.4 ng/mL)	Acute onset of painful swelling and weakness, headache, nausea, and vomiting, DVT, dural sinus thrombosis at superior sagittal sinus, parietal lobar hematoma, brain edema, and midline shift.	Linear purpuric rashes on the right leg	AstraZeneca, 3 days	N/A	None	Vasculitis and erythrocyte extravasation in the dermis and subcutaneous fat tissue	IVIG, methylprednisolone apixaban
Innao et al.[17]	Case report, 1	33, female	TTP	Severe anemia (hemoglobin 68 g/L) with retikulocytosis and critical thrombocytopenia (platelets 1210^9^/L), elevated lactate dehydrogenase (1.280 U/L) and total bilirubin (2.3 mg/dL), and decreased haptoglobin level ( < 0.06 g/L), presence of 3% schistocytes	Asthenia, drowsiness, headache, nausea with abdominal pain	Lower extremity purpura	Pfizer‐BioNTech, first, 9 days	N/A	Nodular Sclerosis classical Hodgkin Lymphoma (NScHL), chemotherapy	N/A	Methylprednisolone, plasma exchange, resh frozen plasma (FFP) Caplacizumab
Jasaraj et al. [18]	Case report, 1	67, female	ITP	PLT = 3000/µL	Bleeding in her gum, subconjunctival hemorrhage in the right eye and hemorrhagic lesions of the tongue and buccal mucosa	Petechial rashes on her legs and chest after the first dose that progressed throughout her body after the second dose,	Pfizer‐BioNTech, both doses, two weeks	Negative	Hypertension, type 2 diabetes mellitus, hypothyroidism, depression, vitamin B12 deficiency, and chronic cluster headaches	N/A	Prednisone, (IVIG) platelets, aminocaproic acid, Rituximab, Eltrombopag
Julian et al. [19]	Case report, 1	72, female	TTP	Platelet count of 12,000/μL, decreasing to 1000/μL	Spontaneous oral bleeding, and headache, multiple episodes of melena	Diffuse petechiae across her arms, legs, and abdomen	Moderna, first, 1 day	N/A	Type 2 diabetes mellitus, and seasonal contact dermatitis	N/A	Dexamethasone, immunoglobulin, aminocaproic acid, rituximab, platelet transfusions
King and Towner [20]	Case report, 1	39, female	ITP	platelet count was 1000/µL	Fatigue and muscle aches	Petechial rash on her trunk, legs, and arms	Pfizer‐BioNTech, second, 3 days	Negative	Polycystic ovary syndrome/norgestimate‐ethinyl estradiol	N/A	Platelets, infusions of immunoglobulin, and methylprednisolone
Kirpalani et al. [21]	Case report, 1	14, female	TTP	Haemolytic anaemia with a haemoglobin of 63 g/L, platelets < 10 × 10^12^/L, bilirubin 68 µmol/L, lactate dehydrogenase (LDH) 626 µ/L, haptoglobin < 0·10 g/L, and the occasional red cell fragment noted on blood film, ADAMTS13 activity testing showing a level of < 1% and ADAMTS13 IgG of 72 µ/mL	Fatigue, headache, confusion	Bruising and ecchymosis	Pfizer‐BioNTech, first, two weeks	Negative	Anxiety, iron deficiency, and postprandial abdominal pain	N/A	Oral prednisone therapeutic plasma exchange (TPE), Rituximab, methylprednisolone, caplacizumab
Mücke et al. [22]	Case report, 1	76, male	Immune complex vasculitis	Elevated blood sedimentations rate, interleukin‐6 levels and C‐reactive protein levels	Symmetric distal limb swelling	Purpuric rash with palpable maculae on extensor and flexor parts of both hands, legs and thighs reaching up to the lower abdomen	Pfizer‐BioNTech, second, 12 days	N/A	Compensated alcoholic liver cirrhosis, NYHA II heart failure, gastrectomy after gastroesophageal junction cancer and prostatectomy after prostate cancer	N/A	Prednisolone
Murali et al. [23]	Case report, 1	95, female	Acquired haemophilia A	Factor VIII level was undetectable at < 0.01 U/mL (range: 0.5 – 1.5 U/mL) and the factor VIII inhibitor level was measured at 5.4	N/A	Widespread ecchymoses especially over the right forearm and left hip, loss of skin integrity overlying a large haematoma on the dorsum of the right hand with resultant bleeding	Pfizer‐BioNTech, first, one week	N/A	Dementia, hypertension, depression, congestive cardiac failure, and a distant history of breast cancer/mirtazapine, frusemide, oxycodone‐naloxone, and paracetamol	N/A	Prednisolone, recombinant Factor VIII and tranexamic acid, rituximab
Osmanodja et al. [24]	Case report, 1	25, male	TTP	ADAMTS‐13 activity returned highly suppressed ( < 1%), with highly elevated ADAMTS‐13 antibodies (72.2 IU/mL)	Headache	Petechiae on both legs	Moderna, first, 4 days	N/A	None	N/A	Plasma exchange fresh frozen plasma, prednisolone daily, caplacizumab
Prasad et al. [25]	Case report, 1	58, male	ITP	platelet count of 3 × 10^9^/L	N/A	Diffuse petechiae along the arms, legs, and abdomen along with numerous oral lesions and gingival bleeding	Moderna, first, 3 weeks	Negative	Hypertension and diabetes	N/A	Dexamethasone, IVIG, fostamatinib
Ruhe et al. [26]	Case report, 1	84, female	TTP	Thrombocytopenia (45 × 10^9^/L), Coombs negative hemolytic anemia (hemoglobin 7.9 g/dL; schistocytes 42‰, haptoglobin < 10 mg/dL; total serum bilirubin 2455 mg/dL; and acute renal failure (serum creatinine 1.95 mg/dL), ADAMTS13 antibodies were reduced but still positive (19.9 U/mL); ADAMTS13 activity was 14%	Multiple subacute emboli without vessel occlusion	Scattered petechiae	Pfizer‐BioNTech, first, two weeks	Negative	N/A	N/A	Rituximab, candesartan
Shonai et al. [27]	Case report, 2	69, male	TTP	Platelet count of 6×10^9^/L	N/A	Oral bleeding and severe purpura	Pfizer‐BioNTech, second, 10 days	N/A	Postoperative intestinal obstruction and hypopharyngeal cancer	N/A	Oral prednisolone
Shonai et al. [27]	Case report, 2	34, female	TTP	And had a platelet count of 3 × 10^9^/L,	Irregular vaginal bleeding	Severe purpura	Moderna, second, 3 weeks	N/A	None	N/A	Oral prednisolone
Sugita et al. [28]	Case report, 1	67, female	Ig A vasculitis	Serum creatinine (sCr) at 0.83 mg/dl, the estimated glomerular filtration rate (eGFR) 52.6 ml/min/1.73m^2^, urine protein (3 + ), protein quantification 5.1 g/gCr, urine occult blood (3 + ), and urine sediment of red blood cells > 100/HPF	Gross hematuria, arthritis, and abdominal pain, leg edema	Purpura on her extremities and trunk	Pfizer‐BioNTech, second, same day,	N/A	Hypertension	Leukocytoclastic vasculitis, suggesting small‐vessel vasculitis.	Methylprednisolone, prednisolone, intravenous cyclophosphamide
Waraich, [29]	Case report, 1	48, male	TTP	Platelet count of 14 × 10^9^/L	Extensive cerebral venous sinus thrombosis (CVST) with subarachnoid haemorrhage, cardiopulmonary arrest, hematuria	Widespread fine petechial rash	AstraZeneca, first, 2 weeks	None	Antibiotic‐treated prostatitis, eczema, asthma and essential hypertension	N/A	IVIG, Thrombectomy, levetiracetam, heparin, argobatran and then fondaparinux.
Wong, [30]	Case report, 2	86, male	ITP	Platelet count of 4 × 10^9^/L	Gingival bleeding, tongue blisters, brain 8 mm left parietal haemorrhage	Widespread ecchymoses	AstraZeneca, N/A, 2 days	Negative	None	N/A	High‐dose dexamethasone, IVIG, platelet transfusion, rituximab
Wong, [30]	Case report, 2	38, female	ITP	Platelet count 3 × 10^9^/L	N/A	Widespread petechiae and purpura	AstraZeneca, N/A, 10 days	N/A	None	N/A	Prednisolone, ivig
Yocum, [31]	Case report, 1	62, female	TTP	White blood cell count 19.25 k/μL, platelets 11 k/μL	Altered mental status	Scattered petechia	Janssen	N/A	Hypertension, hyperlipidemia, hypothyroidism, and gastroesophageal reflux disease	N/A	Hemodialysis, packed red blood cells for anemia, plasma exchange, and methylprednisolone

The review included 35 reported cases of vascular injuries, with 17 (48.57%) males and 18 (51.42%) females. The mean age of the patients was 55.4 years, with a range from 14 to 95 years. The majority of cases manifested symptoms following the first doses of specific vaccines: Pfizer‐BioNTech (*n* = 18, 52.77%), Moderna (*n* = 8, 22.22%), AstraZeneca (*n* = 8, 22.22%), and Janssen (*n* = 1, 2.77%).

The interval between vaccination and the onset of symptoms varied significantly, ranging from less than 1 day to 3 weeks. This variability suggests differing individual responses to vaccination and underscores the importance of monitoring patients for a range of symptoms during this timeframe.

Patients were diagnosed with several conditions related to vascular injury: thrombocytic thrombotic purpura (TTP) in 15 cases (42.86%), immune thrombocytopenic purpura (ITP) in 12 cases (34.28%), vasculitis in 5 cases (14.28%), and acquired hemophilia A in 3 cases (8.57%). Importantly, none of the patients tested positive for SARS‐CoV‐2 at the time of evaluation, suggesting that these conditions were unrelated to active COVID‐19 infection.

Cutaneous symptoms were notably prevalent among the patients, with petechiae observed in 17 cases (48.57%), purpura in 11 cases (31.42%), and ecchymosis in 6 cases (17.14%). These manifestations primarily affected the lower extremities, indicating a possible link between the vaccinations and localized vascular responses.

The characteristics of all included studies are summarized in Table 1, detailing patient demographics, types of vascular injuries, and relevant clinical findings. Importantly, statistical hypothesis testing was avoided in favor of presenting effect sizes and precise estimates to convey more meaningful clinical insights. Where appropriate, *p* values are reported following standard conventions: for instance, *p* < 0.001 for highly significant findings or exact values to the nearest thousandth or hundredth as relevant. This approach ensures transparency and clarity while avoiding overreliance on statistical testing.

This comprehensive summary highlights the implications of vascular injuries following COVID‐19 vaccination. Further research is warranted to explore the underlying mechanisms and long‐term outcomes associated with these rare but significant adverse events.

## 4 Discussion

Vaccination is crucial for the prevention of infectious diseases, and studies have indicated that COVID‐19 vaccines significantly reduce morbidity and mortality among vaccinated individuals. However, it is important to recognize that COVID‐19 vaccines can be associated with various dermatological adverse effects, some of which may lead to critical and life‐threatening conditions.

Patients presenting to dermatology clinics with complaints of petechiae, purpura, and ecchymosis should undergo a thorough clinical evaluation. Physicians are advised to inquire specifically about bleeding symptoms, such as hematuria, vaginal bleeding, and melena. Additionally, unstable vital signs or any alteration in mental status necessitate prompt medical intervention [9, 10, 11, 12, 13, 14, 15].

In the context of COVID‐19, both petechial exanthems and cutaneous leukocytoclastic vasculitis have been well‐documented. One hypothesis suggests that the presence of viral proteins in the endothelial layer of dermal vessels may induce the development of petechiae and purpura. In cases of vasculitis, immune complex accumulation activates the complement cascade, leading to damage of small vessel walls. Notably, patients with COVID‐19 can exhibit elevated pro‐coagulant factors alongside thrombocytopenia, although the precise pathogenesis of this phenomenon remains incompletely understood.

Our study found that most cases presented with petechiae on the lower extremities, with subsequent diagnoses of TTP and ITP. Vaccines have the potential to activate the immune system, which may trigger the development of autoimmune disorders such as ITP and TTP. The majority of cases of ITP following COVID‐19 vaccination have been linked to mRNA vaccines. This finding aligns with our results, which showed a predominance of cases following Pfizer‐BioNTech and Moderna vaccinations.

Secondary ITP has been associated with various vaccines, and one hypothesis posits that it may arise due to molecular mimicry postvaccination. Additionally, the theory of Autoimmune/Inflammatory Syndrome Induced by Adjuvants (ASIA) suggests that vaccine adjuvants can trigger a spectrum of immune responses. In cases of severe thrombocytopenia, where platelet counts fall below 30,000, patients are at a higher risk of mucocutaneous bleeding and life‐threatening hemorrhages. Our study recorded instances of ITP following COVID‐19 vaccination characterized by significantly low platelet counts, alongside cutaneous manifestations and bleeding from other sites, such as epistaxis and subconjunctival hemorrhage [15, 16, 17, 18, 19, 20]. Notably, we documented a case of severe ITP in a 63‐year‐old female patient who presented with a platelet count of zero after receiving the second dose of the Pfizer‐BioNTech vaccine [13].

TTP has also been reported following various vaccinations. In the literature, the time interval between influenza vaccination and the onset of TTP ranges from 5 to 14 days. Cases of TTP have been reported 15 days after rabies vaccination and following the administration of the 23‐valent pneumococcal polysaccharide vaccine [20, 21, 22, 23, 24, 25, 32]. In our study, the time interval between vaccination and TTP occurrence varied from 1 day to 3 weeks. One theory regarding this phenomenon is the notion of vaccine‐induced thrombosis with thrombocytopenia (VITT), which suggests that vaccine antigens may trigger an immune response against the ADAMTS13 gene, leading to the production of inhibitors against this enzyme via cross‐reactivity with polysaccharide antigens or adjuvants [10, 11]. Our findings included severe cases of TTP that resulted in life‐threatening conditions, such as extensive cerebral venous sinus thrombosis (CVST) with subarachnoid hemorrhage and multiple subacute emboli without vessel occlusion [26, 27, 28].

Additionally, we observed severe cases of IgA vasculitis, which were generally managed with corticosteroids. IgA vasculitis has also been reported following measles, mumps, rubella (MMR), or influenza vaccinations. Furthermore, our study identified cases of acquired hemophilia A, which presented with severe ecchymosis following COVID‐19 vaccination. Acquired hemophilia A is a rare condition associated with a high morbidity and mortality rate of approximately 21% [11]. In this disorder, autoantibodies against coagulation factor VIII are formed. All cases of acquired hemophilia A in our study were linked to mRNA vaccines and were managed with corticosteroids and factor VIII replacement.

In conclusion, while COVID‐19 vaccines play a critical role in public health, healthcare providers must remain vigilant regarding the potential for rare but serious dermatological adverse effects. Further research is needed to elucidate the underlying mechanisms and to improve patient management strategies for those experiencing cutaneous manifestations postvaccination.

## 5 Conclusion

COVID‐19 and COVID‐19 vaccines have been associated with various side effects. Our study suggests a potential, though not definitively established, role of COVID‐19 vaccines in the development of petechiae, purpura, and ecchymosis. These manifestations require further investigation, as they may indicate critical and life‐threatening conditions that necessitate prompt treatment. Most patients exhibited symptoms following the first dose of mRNA vaccines (Pfizer‐BioNTech and Moderna). The majority of cases presented with petechiae on the lower extremities, which were subsequently diagnosed as thrombotic thrombocytopenic purpura (TTP) or immune thrombocytopenic purpura (ITP). We recommend conducting large randomized controlled trials (RCTs) to evaluate the possible association between COVID‐19 vaccines and these conditions.

## Author Contributions

**Yasamin Kalantari:** conceptualization, investigation. **Seyed Mohamad Sadegh Mirahmadi:** writing – original draft, methodology. **Sanam Alilou:** validation, visualization. **Sara Sadeghi:** writing – review and editing, software. **Zeinab Aryanian:** formal analysis, data curation. **Alireza Jafarzadeh:** writing – review and editing; writing – original draft. **Azadeh Goodarzi:** project administration, supervision, resources.

## Ethics Statement

This project was approved by the Ethics Committee of Iran University of Medical Sciences with the title: “*A Systematic Review of Vascular Injuries: A Review of Petechiae, Purpura, and Ecchymosis in Critical Situations Following COVID‐19 Vaccination*”, with the ethical code IR.IUMS.FMD.REC.1403.124, date of approval: 2023‐11‐16.

## Consent

The authors obtained consent to publish. The current manuscript contains no individual person's data. Therefore, consent to publish is not applicable.

## Conflicts of Interest

The authors declare no conflicts of interest.

## Transparency Statement

The lead author Azadeh Goodarzi affirms that this manuscript is an honest, accurate, and transparent account of the study being reported; that no important aspects of the study have been omitted; and that any discrepancies from the study as planned (and, if relevant, registered) have been explained.

## Data Availability

The data that support the findings of this study are available from the corresponding author upon reasonable request.
